# Pre- and Procedural Factors Influencing the Success of In Vitro Fertilization: Evaluating Embryo Quality and Clinical Pregnancy in Cases of Tubal Factor Infertility

**DOI:** 10.3390/jcm13195754

**Published:** 2024-09-27

**Authors:** Zoltan Kozinszky, Kristóf Bereczki, Viktor Vedelek, Petra Bicskei, Mariann Tabi, Csaba Ekes, Noémi Lajkó, Olga Nagy, Rita Sinka, Anna Vágvölgyi, János Zádori

**Affiliations:** 1 Capio Specialized Center for Gynecology, Solna, 182 88 Stockholm, Sweden; 2 Department of Obstetrics and Gynecology, Albert Szent-Gyorgyi Medical School, University of Szeged, 6725 Szeged, Hungary; bereczkikristof9@gmail.com (K.B.); nagy.olga.maria@med.u-szeged.hu (O.N.); 3 Department of Genetics, Faculty of Science and Informatics, University of Szeged, 6726 Szeged, Hungaryrsinka@bio.u-szeged.hu (R.S.); 4 Institute of Reproductive Medicine, Albert Szent-Gyorgyi Medical School, University of Szeged, 6723 Szeged, Hungary; bicskei.petra@med.u-szeged.hu (P.B.); tabi.mariann@med.u-szeged.hu (M.T.); ekes.csaba@med.u-szeged.hu (C.E.); zadori.janos@med.u-szeged.hu (J.Z.); 5 Department of Internal Medicine, Albert Szent-Gyorgyi Medical School, University of Szeged, 6725 Szeged, Hungary; vagvolgyi.anna@med.u-szeged.hu

**Keywords:** in vitro fertilization, clinical pregnancy, fallopian tube blockage, non-tubal factor infertility

## Abstract

**Introduction**: While tubal occlusion is a prevalent cause of infertility, accounting for 11–35% of infertility cases among women, there remains a limited understanding of the factors influencing clinical pregnancy following in vitro fertilization (IVF). **Methods**: In our retrospective, cross-sectional cohort study conducted at a single tertiary center, medical records of women aged 19 to 43 years were analyzed. Logistic regression models were employed to identify the prognostic factors associated with clinical pregnancy after IVF in patients with tubal factor infertility, excluding cases with hydrosalpinx. **Results**: Data from 219 women diagnosed with tubal occlusion were compared to 1140 cases with non-tubal indication, covering a total of 1359 IVF cycles. A lower maternal age (adjusted odds ratio [AOR]: 0.89, *p* = 0.001) and a higher embryo quality (AOR: 1.26, *p* = 0.01) emerged as important factors in clinical pregnancy in the tubal infertility group. Moreover, a lower maternal (AOR:0.91, *p* < 0.01) and paternal age (*p* = 0.001), and favorable semen quality (AOR: 1.32, *p* = 0.03) were critical determinants in the non-tubal infertility group. BMI was generally higher in tubal infertility patients (*p* = 0.01). Furthermore, FSH level (AOR: 0.93, *p* = 0.004), AMH level (*p* < 0.04), number of embryos transferred (AOR: 2.04, *p* < 0.001), and embryo quality (AOR: 1.26, *p* < 0.001) came into prominence only in the non-tubal infertility group. The clinical pregnancy rate (34.2%) of women with tubal occlusion did not differ significantly from those in other forms of infertility undergoing IVF (35.4%). **Conclusions**: Although tubal infertility is typically anticipated to yield the highest clinical pregnancy rates following IVF, it is crucial to acknowledge that both maternal and paternal characteristics can also significantly impact the outcomes.

## 1. Introduction

Several causes of infertility, such as male factor infertility, tubal occlusion, reduced ovarian reserve, and other conditions unresponsive to less invasive treatments, can be effectively addressed through in vitro fertilization (IVF). A variety of pre-procedural factors can influence the success of IVF, with the primary determinant being the biological age of the individual supplying the oocytes [1]. Other major factors that can significantly impact IVF outcomes include poor ovarian reserve, the presence of hydrosalpinx, and the use of substances, e.g., tobacco [2]. Hydrosalpingeal fluid can hinder the establishment of a successful pregnancy through various adverse effects, including mechanical interference, the presence of microorganisms and endotoxins, altered cytokine levels, nutrient deficiencies, and oxidative stress [3]. However, at least one meta-analysis has identified evidence indicating a reduction in ovarian reserve following salpingectomy [4]. Other pre-procedural factors, such as leiomyomas and endometriomas, lack a clear consensus on the optimal approach for treatment [5]. While obesity has moderate impacts on IVF outcomes, it is imperative that overweight or obese patients are not excluded from accessing IVF care. This should be contingent upon receiving comprehensive counselling, undergoing necessary pretreatment protocols, and participating in shared decision-making processes [6]. Both major and minor female pre-procedural factors, in conjunction with male partner factors such as semen quality and paternal age, collectively determine the efficacy of the in vitro fertilization procedure. The purpose of this study was to further elucidate these IVF-linked factors and to determine whether tubal occlusion without hydrosalpinx, or following salpingectomy, has a detrimental effect on IVF outcomes.

## 2. Goals

Our objective was to examine the relationship among anamnestic parameters, endometrial thickness, embryo quality, nutritional status as expressed by body mass index (BMI), and clinical pregnancy in women conceived IVF/ICSI treatment with tubal factor infertility without hydrosalpinx, compared to non-tubal infertility cases.

## 3. Methods

A retrospective cohort study was carried out in the Institute of Reproductive Medicine, University of Szeged. The study included data abstracted from the medical database on women aged 19 to 43 years who were treated for IVF with or without intracytoplasmic sperm injection (ICSI) with successful oocyte retrieval between 1 January 2020 and 30 September 2023.

All couples (*n* = 1554) had established infertility diagnosis after detailed sonographic and laboratory examinations. Furthermore, the occlusion of the fallopian tube was diagnosed with hysterosalpingography (HSG). HSG was scheduled, preferably in the initial phase of infertility. HSG was conducted by injecting a water-soluble contrast medium via a cannula or balloon catheter. Women with unilateral or bilateral hydrosalpinx were subjected to uni- or bilateral salpingectomy carried out by laparoscopy and were considered also to have tubal factor infertility. If sonographic findings indicated a space-occupying, intrauterine pathology (septated or subseptated cavity, submucosal fibroid, polyp) in the uterine cavity, then the woman was referred to a hysteroscopic operation.

Only couples with all available clinical data were recruited in the study. In addition, each couple was represented with the pregnancy outcome following the latest treatment. Repeat analyses of couples were not noticed during the study period. The following characteristics of all subjects were abstracted from the medical database: age (years), weight (kg), height (cm), endometrium development at embryo transfer (ET) (mm), follicle-stimulating hormone (FSH; IU/L), luteinizing hormone (LH; IU/L), and anti-Müllerian hormone (AMH; ng/mL). The BMI collected at the initial visit during IVF/ICSI treatment was calculated as the body weight (kg) divided by the square of the height (m^2^). Women were divided based on the World Health Organization [7] classification as underweight (BMI ˂ 18.5 kg/m^2)^, normal weight (BMI: 18.5–24.9 kg/m^2^), overweight (BMI: 25–29.9 kg/m^2^), and obesity (BMI > 30 kg/m^2)^ with a combination of the categories of class I, II, and III obesity into a single obesity category in order to simplify the statistical analyses. The study evaluated several factors related to infertility and IVF/ICSI treatment, including the duration of infertility (in years), subfertility due to a single tubal factor versus non-tubal factors, duration of ovarian stimulation (in days), number of discontinued cycles, follicle count, and the total number of embryos transferred (single versus double ET; SET vs. DET). Additionally, the stimulation protocol (gonadotropin-releasing hormone [GnRH] agonist—ultrashort, short, long—or GnRH antagonist) and the fertilization method of the transferred embryos (IVF, ICSI, or a combination in DET cases) were recorded. On the andrological side, key factors such as the partner’s age (in years) and various semen analysis parameters were documented, including sperm concentration (×10^6^/mL), sperm motility (percentage of moving sperm), and morphology (percentage of sperm with a normal shape and structure). Semen parameters that fell within the normal reference range (normospermia) were evaluated separately.

### IVF/ICSI Stimulation Protocol

Taking into account the ovarian function, sex hormone levels, age, and weight of each woman, individualized ovarian stimulation protocols were employed, primarily utilizing a flexible GnRH-antagonist approach, with GnRH-agonist protocols used less frequently. Cycle selection occurred on the second or third day. The initial consultation involved an ultrasound examination (Samsung Medison HS50; endovaginal probe: EVN4-9, 4–9 MHz), assessment of the antral follicle count, and measurement of FSH, LH, prolactin, and thyroid-stimulating hormone levels. FSH treatment started on the 2nd–3rd day of menstruation, and it was adjusted according to patient response, who was monitored by serial transvaginal sonography and estrogen (E2) level from day five and every 2–3 days on. When the leading follicles reached a diameter of 14 mm, a daily dose of 0.25 mg GnRH antagonist was administered until the day of ovulation triggering. The follicular rupture was induced using hCG once the patient had at least three follicles measuring ≥18 mm. Oocyte retrieval was performed transvaginally 36 h following hCG (Ovitrelle^®^, Merck KGaA, Merck Serono, Darmstadt, Germany) administration. The laboratory and ET processes were standardized across all cycles and adhered to established protocols. Follicular fluid was collected in pre-warmed 14 mL round-bottom tubes (Thermo Fisher Scientific Inc., Roskilde, Denmark) and maintained in a heating block set to 37 °C. Oocyte identification took place under laminar flow using 90 mm petri dishes (Thermo Fisher Scientific Inc., Roskilde, Denmark). The cumulus–oocyte complexes were retrieved into Nunc IVF Center Well Dishes (Thermo Fisher Scientific Inc., Roskilde, Denmark) containing G-MOPS PLUS medium (Vitrolife AB, Gothenburg, Sweden). The complexes were subsequently transferred to 5-well dishes (Vitrolife AB, Gothenburg, Sweden) containing G-IVF medium (Vitrolife AB, Gothenburg, Sweden) under an oil overlay (Hypure Heavy Ovoil, Kitazato Corp., Shizuoka, Japan) and incubated under controlled conditions of 37 °C, 6% CO_2_, and 5% O_2_ (K-Systems G210 InviCell, CooperSurgical, Birkerød, Denmark, and PLANER Benchtop Incubator BT37, (CooperSurgical Inc., Birkerød, Denmark). Fertilization was carried out 2–4 h after oocyte retrieval, either by conventional IVF or ICSI, with the choice primarily determined by semen quality parameters. For conventional IVF, only fresh semen samples with normal parameters (normozoospermia) were used to fertilize cumulus–oocyte complexes, which were placed in 5-well dishes containing G-IVF media under an oil overlay. For ICSI, the oocytes were denuded using hyaluronidase (80 IU/mL; SynVitro Hyadase, Origio, Ballerup, Denmark) before injection. Following fertilization, the zygotes were cultured in G1 and G2 media (Vitrolife AB, Gothenburg, Sweden) within 5-well dishes inside incubators set at 6% CO_2_, 5% O_2_, and 37 °C (K-Systems G210 InviCell, CooperSurgical Inc., Birkerød, Denmark; PLANER Benchtop Incubator BT37, CooperSurgical Inc., Birkerød, Denmark). At 17–20 h post-insemination, zygotes were evaluated for the presence of two pronuclei, representing the maternal and paternal genetic contributions. Zygotes showing more than two pronuclei were classified as abnormal and discarded. The highest-quality embryos, either at the cleavage or blastocyst stage, were selected for transfer. Only blastocyst-stage embryos were cryopreserved on days 5 or 6 of culturing. Embryo quality was assessed based on the number of blastomeres and the percentage of fragmentation [8]. Prior to transfer, blastocysts were placed in EmbryoGlue (Vitrolife AB, Gothenburg, Sweden) for 20–40 min. Embryo transfers were performed using the Wallace Embryo Replacement Catheter (soft, 18 cm, Cooper Surgical Inc., Trumbull, Connecticut, CT, USA), and two-dimensional transvaginal ultrasound (Samsung Medison HS50; Samsung Electronics Co., Ltd./Samsung Medison Co., Ltd., Gangwon, Republic of Korea, endocavitary probe: EVN4-9, 4–9 MHz) was utilized for both follicular punctures and embryo transfers. Endometrial thickness (mm) was measured using the same ultrasound equipment on the day of ET, with the measurement taken from the outer edge of the endometrial-myometrial junction to the outer edge of the widest part of the endometrium [9].

A customized evaluation system was developed to standardize differences in embryo development and quality across various transfer days. This system utilized a specific embryo scoring method for statistical analysis, with further details provided in our previous publication [6]. In summary, the morphological embryo grading system introduced by Gardner et al. [10] offers a reliable representation of embryo quality, ensuring standardization in assessments. The quality of embryos plays a critical role in determining the success of IVF treatment cycles. Embryo expansion is quantified on a scale from 1 to 6, while the trophectoderm and inner cell mass are graded using an A-B-C classification. The combination of these characteristics produces 54 potential embryo quality grades. Based on this embryo, grades were grouped into the following three categories: “excellent, good, or poor” [11]. To utilize embryo quality alignment for both single and double ET (SET and DET, respectively), concomitantly, embryo score composite was introduced as a new variable. Embryo score composite was interpreted by the following categories: SET with “poor” embryo quality was marked with 1; DET with “poor” + “poor” was marked with 2; DET with “poor” + “good” was marked with 3; DET with “poor” + “excellent” was rated as 4; SET with “good” was marked with 5; DET with “good” + “good” was categorized as 6, SET with excellent was interpreted by 7, and DET with “excellent” + “excellent” was displayed as category 8.

## 4. Statistical Analyses

Statistical analyses were performed using SPSS version 23 (IBM Corp., Armonk, NY, USA). Continuous variables were reported as mean ± standard deviation (SD), while categorical variables were presented as frequencies and percentages. The parametric distribution of continuous variables was verified by the low standard deviations of kurtosis and skewness. Any type of pregnancy, as a category of pregnancy outcome, was the major exposure variable.

Univariate comparisons between pregnancy outcome measurements (pregnancy vs. failure of conception) subgroups for both tubal and non-tubal factor infertility were assessed with the independent samples *t*-test for continuous variables. Categorical variables were compared across subgroups using chi-square tests. Crude odds ratios (OR) and Cornfield 95% confidence intervals (CIs) were presented. The resultant ORs for tubal factor and non-tubal factor infertility were compared using Mantel–Haenszel tests, affording an approximation of the treatment effect of tubal factor infertility on the likelihood of clinical pregnancy. The effect of infertility type on the chance of pregnancy was assessed by employing two-way ANOVA for continuous variables.

Binary logistic regression was utilized to assess how the independent variables influence the pregnancy outcome measurement (any type of pregnancy), with the interrelations being controlled for confounders. All maternal, paternal, obstetric history, and IVF/ICSI-related characteristics, as well as variables associated with infertility, were represented as independent factors. All factors in the multivariate analyses were adjusted for maternal age, BMI, and previous number of IVF/ICSI cycles, as these factors determine fertility chances per se. Furthermore, multivariable logistic regression was performed to evaluate the predictors determining any type of pregnancy in both tubal factor infertility and other types of infertility separately. The group of no clinical pregnancies served as a reference. Multivariable dependency of the outcome variable on both categorical and continuous variables was assessed through stepwise logistic regression (forward selection), utilizing the likelihood ratio criterion (P_in_ = 0.05; P_out_ = 0.10). Multivariable regression was applied to variables that demonstrated significant or near-significant differences in the univariate analysis. Besides *p*-values, the adjusted OR was also calculated with a 95% CI for multivariable logistic regression. A two-tailed significance threshold of 5% was employed, and *p*-values were adjusted for multiple comparisons using the Holm–Bonferroni method.

The Pearson correlation coefficient was computed using the pandas. DataFrame.corr method in Python 3.0 to assess the correlation. The Seaborn library was used to generate heatmaps.

## 5. Results

### Clinical Characteristics of the Women Receiving IVF/ICSI Presented by Clinical Pregnancy

All cases of tubal factor infertility combined with another type of infertility (*n* = 165) were excluded from the analyses, and the final cohort included 1389 women. A total of 219 women had exclusively tubal occlusion, while 1140 couples had other than tubal factor infertility.

As pregnancy outcome, the occurrence of live births [singleton (*n* = 390) or twin pregnancy (*n* = 38)] or the lack of conception [no pregnancy (*n* = 881)] were recorded. Biochemical pregnancy (*n* = 25), clinical miscarriage (*n* = 23), and ectopic pregnancy (*n* = 2) [12] were also registered, and patients with these outcomes were also included in any type of pregnancy group. During the study period, clinical pregnancy (34.2%) followed infertility associated with only tubal occlusion, which was not significantly different from those who had non-tubal infertility (35.4%). The anamnestic, biometric characteristics, and infertility-related factors in relation to any type of pregnancy are shown in Table 1. ORs showed that a lower mean age was associated with a higher prevalence of any type of pregnancy. After adjusting for all selected covariates, maternal age was found to be inversely associated with the odds of clinical pregnancy [adjusted odds ratio (AOR): 0.89 and 0.91] in both tubal and non-tubal groups. Subjects who became pregnant were slightly thinner in the tubal infertility group, whereas similar weights were measured in the non-tubal infertility subgroups, and this tendency demonstrated a significant difference between the two infertility clusters. No one BMI category (underweight, normal weight, overweight, obesity) showed any significant relation to pregnancy. Occurrence of any type of previous pregnancy, duration of infertility, number of discontinued cycles, LH levels, and duration of stimulation were not relevant factors for pregnancy. A smaller number of previous IVF/ICSI did not predict pregnancy either in the univariate or in the multivariate analyses among women with tubal infertility (AOR: 0.97), but it came into prominence as a predictor of conception in non-tubal infertility (AOR: 0.87). Similarly, lower levels of FSH (AOR: 0.93) and higher AMH levels (AOR: 1.03) prognosticated a higher odd for pregnancy in the non-tubal infertility group but not in the tubal occlusion group (AOR: 0.93 and AOR: 1.04, respectively). Infertile women are more likely to become pregnant following DET, and this is significantly more pronounced among women diagnosed with non-tubal infertility (AOR: 2.04 vs. 1.75). Generally, the higher the embryo score, the higher the odds of pregnancy, and the embryo score composite has a comparable effect in both the tubal and non-tubal infertility groups. A lower paternal age represented a significantly higher odd for pregnancy among women with non-tubal pregnancy, but not in the tubal blockage group. Sperm concentrations were higher in the tubal infertility group, and the difference between pregnant and non-pregnant subgroups was more noticeable in this group. A negligible difference in normal sperm morphology was observed in favor of non-pregnant couples in the tubal infertility group, whereas a minimal, non-significant opposite tendency could be described in the non-tubal group. The rate of male partners with normospermia did not influence the chance of pregnancy among women with tubal infertility, but normospermia indicated significantly a higher odd for pregnancy among women with non-tubal infertility.

The significant independent prognosticating factors for both clinical pregnancy and infertile couples are detailed in Table 2. The occurrence of pregnancy was significantly dependent on the maternal age (AOR: 0.87) and the number and quality of transferred embryos (AOR: 1.24) in cases of tubal occlusion. Apart from these factors, a lower number of IVF/ICSI cycles performed previously (AOR: 0.89) and a lower FSH level (AOR: 0.93) detected during the present IVF/ICSI increased the likelihood of conception in the non-tubal group.

**Figure 1:**
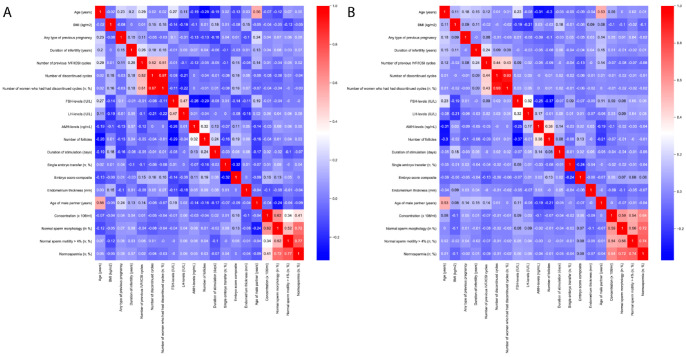
Heatmaps represent Pearson correlation coefficients. (**A**) Correlations in “tubal occlusion” group (*n* = 219) and (**B**) correlations in “non-tubal occlusion” group (*n* = 1140).

Figure 1 demonstrates the correlation heatmaps within the tubal factor and non-tubal infertility groups. The heatmaps show similar trends in both tubal occlusion (Figure 1A) and non-tubal occlusion (Figure 1B) groups. Intermediate and higher correlations can be observed regarding parental age, cycle numbers (IVF/ICSI, discontinued), FSH and LH levels, and male factors. Considerable differences (≥0.2) between the two groups can be found in the correlation of sperm concentration with normospermia and sperm motility. Additionally, LH levels and embryo score correlation with discontinued cycle numbers also show different patterns.

## 6. Discussion

### Clinical Characteristics of the Women Conceived IVF/ICSI Presented by Clinical Pregnancy

In our study, the clinical pregnancy rate (34.2%) associated with infertility due to tubal occlusion was not significantly different from that in non-tubal infertility cases (35.4%). Therefore, tubal factor infertility, excluding cases caused by hydrosalpinx, was not proven to be superior in clinical pregnancy following IVF compared to other types of infertility. This finding also questions the appropriateness of using patients with tubal factor infertility as a control group in studies. A lower maternal mean age was associated with a higher prevalence of any type of pregnancy, which is consistent with the literature [13]. In line with this finding, after adjusting for all selected covariates, maternal age was found to be inversely associated with the odds of clinical pregnancy, with similar odds observed in both tubal and non-tubal infertility groups. There was no statistically significant variance in BMI observed between the pregnant and non-pregnant subgroups of individuals with tubal factor infertility, as well as those with non-tubal factor infertility. This finding is consistent with our previous work [6], where none of the presented BMI categories showed a statistically significant relationship with clinical pregnancy as an IVF outcome.

A lower number of previous IVF/ICSI cycles did not predict pregnancy in either univariate or multivariate analyses among women with tubal infertility, but emerged as a significant predictor of conception in cases of non-tubal infertility. This finding aligns with a recent retrospective analysis by Fang et al. [14] involving 13,172 patients who underwent 16,975 IVF/ICSI-ET cycles. The study showed that previous embryo implantation failures independently affect clinical pregnancy; as the number of failures increases, both rates decrease significantly. Similarly, lower FSH levels and higher AMH levels predicted a higher likelihood of pregnancy in the non-tubal infertility group but not in the tubal occlusion group. Elevated basal FSH levels indicate diminished ovarian reserve [15]. AMH is commonly used to assess ovarian reserve in women undergoing IVF/ICSI treatment and has been utilized to predict pregnancy outcomes after assisted reproduction [16]. Numerous studies [17,18,19] and a meta-analysis [20] have reported a positive correlation between AMH levels and pregnancy rates following IVF/ICSI treatment. The fact that these associations were not confirmed in the tubal occlusion group is noteworthy and supports the hypothesis that tubal infertility behaves uniquely and may not be an ideal choice as a control group.

It is known that a single SET cycle results in lower birth rates than a single DET cycle [21]. In the present study, infertile women were more likely to achieve pregnancy following DET, and this effect was significantly greater among those diagnosed with tubal infertility. This trend is certainly noteworthy, and to the best of our knowledge, a similar observation has not yet been documented.

Not surprisingly, a higher embryo score was generally associated with increased odds of pregnancy, and the composite embryo score had a comparable impact in both tubal and non-tubal infertility groups. In our previous work [6], we developed a support vector machine that achieved an average prediction accuracy of 61.71% using 22 dimensions of medical data, identifying maternal age and embryo quality as the most significant predictors.

From the male perspective, a lower paternal age was associated with significantly higher odds of clinical pregnancy among women with non-tubal infertility, but not in the tubal blockage group. The impact of paternal age beyond maternal age has been indicated on clinical pregnancy [22,23]. A meta-analysis, which included a total of 32,484 cycles from 16 autologous oocyte studies, noted a statistically significant effect of paternal age <40 years on clinical pregnancy [24]. The rate of male partners with normospermia did not influence the chance of pregnancy among women with tubal infertility. However, normospermia significantly increased the odds of pregnancy among women with non-tubal infertility.

Clearly, there is no compelling evidence that assisted reproductive technology (ART) outcomes are dependent on sperm parameters. This result could be immediately transposed into clinical practice, aiding in the selection of the most effective ART approach for infertile couples or recommending therapies for the male partner to enhance sperm number, motility, and morphology. An increasing body of research suggests that therapeutic interventions can enhance sperm quantity and quality, as well as overall male health, ultimately leading to improved reproductive outcomes, even when ICSI is the only available option [25]. The differing effects of normozoospermia between the two groups require further evaluation and analysis with a larger sample size. Although paternal age and sperm quality have been shown to significantly influence IVF/ICSI outcome. It is important to emphasize that both the age of the female partner and the age of the male partner play crucial roles in determining the success of the IVF/ICSI procedure.

## 7. Conclusions

In summary, our study found that the clinical pregnancy rate associated with tubal factor infertility was not significantly different from that of non-tubal infertility cases, challenging the notion that tubal infertility leads to superior IVF/ICSI outcomes. Maternal age was a key factor in predicting clinical pregnancy in both groups, with younger age increasing the odds of pregnancy. Notably, factors such as FSH and AMH levels, which typically correlate with pregnancy success in non-tubal cases, were not significant predictors in the tubal occlusion group. There was no statistically significant variance in BMI observed between the clinically pregnant and non-pregnant subgroups for both tubal and non-tubal infertility cases. Additionally, DET was more effective in achieving pregnancy in tubal infertility cases, a finding not previously documented. These results suggest that tubal factor infertility behaves uniquely and may not be suitable as a control group in studies, highlighting the need for further research into its distinct characteristics. Our study was conducted in a single center providing ubiquitous clinical management, ensuring a larger treatment effect. One of our unique findings is that embryo quality is a more prominent factor in both single and double ET than the number of embryos. Primarily, a higher morphological embryonal phenotype is encountered with a greater clinical pregnancy rate, irrespective of the number of the transferred embryo.

Our study has certain limitations, primarily due to its retrospective design. Another limitation is the relatively small sample size in the group of patients with tubal factor infertility, which may have limited the ability to detect statistically significant differences.

## Figures and Tables

**Table 1:** Clinical phenotype of the women conceived IVF/ICSI presented by clinical pregnancy at the Institute of Reproductive Medicine, Albert Szent-Györgyi Medical Centre, University of Szeged, Szeged, Hungary between 1 January 2020 and 30 September 2023 regarding clinical pregnancy (*n* = 1359).

	Only Tubal Factor Infertility (*n* = 219)								Non-Tubal Factor Infertility (*n* = 1140)								*p*-Value ^c^
	Any Type of Pregnancy (*n* = 75)		No Pregnancy (*n* = 144)		*p*-Value ^a^	Unadjusted OR (95% CI) ^a^	*p*-Value ^b^	Adjusted OR (95% CI) ^b^	Any Type of Pregnancy (*n* = 403)		No Pregnancy (*n* = 737)		*p*-Value ^a^	Unadjusted OR (95% CI) ^a^	*p*-Value ^b^	Adjusted OR (95% CI) ^b^	
	*n*	%	*n*	%					*n*	%	*n*	%					
Clinical data																	
Maternal age (years)	33.45 ± 4.18		35.76 ± 4.49		<0.001	1.08–3.54	0.001	0.89 (0.83–0.95)	33.91 ± 4.52		35.97 ± 4.80		<0.01	1.48–2.62	<0.001	0.91 (0.89–0.94)	0.36
BMI (kg/m^2^)	25.33 ± 4.85		26.06 ± 5.20		0.32	−0.70–2.16	0.28	0.97 (0.92–1.03)	24.69 ± 5.16		24.69 ± 5.14		0.99	−0.62–0.63	0.54	1.01 (0.98–1.03)	0.01
Any type of previous pregnancy	35	46.7	76	52.8	0.40	0.78 (0.45–1.37)	0.95	0.98 (0.54–1.79)	123	32.8	258	35.0	0.47	0.90 (0.70–1.14)	0.41	0.89 (0.68–1.17)	0.32
In vitro fertilization related factors																	
Duration of infertility (years)	4.23 ± 2.90		4.77 ± 2.95		0.20	−0.28–1.36	0.45	0.96 (0.86–1.07)	4.16 ± 2.98		4.41 ± 3.08		0.18	−0.12–0.62	0.91	1.00 (0.96–1.05)	0.36
Number of previous IVF/ICSI cycles	1.88 ± 1.17		2.09 ± 1.47		0.29	−0.18–0.60	0.79	0.97 (0.76–1.23)	1.76 ± 1.07		2.05 ± 1.35		˂0.001	0.13–0.44	0.002	0.84 (0.76–0.94)	0.42
Number of discontinued cycles	0.12 ± 0.37		0.12 ± 0.34		0.97	−0.10–0.10	0.79	1.15 (0.42–3.12)	0.17 ± 0.40		0.15 ± 0.42		0.54	−0.03–0.07	0.34	1.19 (0.84–1.69)	0.42
Number of women who had had discontinued cycles (*n*; %)	8	10.6	16	11.1	1.00	0.96 (0.34–2.35)	0.93	1.05 (0.35–3.18)	47	11.7	99	13.4	0.41	0.85 (0.59–1.23)	0.53	1.15 (0.75–1.76)	0.46
FSH level (IU/L)	6.94 ± 3.40		7.91 ± 3.53		0.05	−0.01–1.95	0.17	0.93 (0.83–1.03)	7.21 ± 2.66		8.11 ± 3.12		˂0.001	0.56–1.25	0.004	0.93 (0.89–0.98)	0.32
LH level (IU/L)	5.59 ± 2.54		5.71 ± 2.33		0.71	−0.55–0.80	0.84	0.99 (0.87–1.12)	5.96 ± 2.93		6.11 ± 2.80		0.40	−0.20–0.50	0.22	0.97 (0.93–1.02)	0.07
AMH level (ng/mL)	2.40 ± 1.57		2.09 ± 1.71		0.20	−0.78–0.16	0.65	1.04 (0.87–1.24)	2.83 ± 2.38		2.37 ± 2.26		0.001	−0.74-−0.18	0.31	1.03 (0.97–1.09)	0.04
Number of follicles	8.16 ± 3.86		7.33 ± 3.73		0.12	−1.90–0.23	0.75	1.01 (0.94–1.10)	8.59 ± 3.90		7.70 ± 3.75		<0.01	−1.35–−0.43	0.19	1.02 (0.99–1.06)	0.18
Duration of stimulation (days)	10.13 ± 2.07		10.12 ± 1.79		0.96	−0.55–0.52	0.61	0.96 (0.81–1.13)	10.28 ± 1.79		10.28 ± 2.14		0.96	−0.24–0.25	0.59	0.98 (0.92–1.05)	0.32
Single embryo transfer (*n*; %)	21	28	58	40.3	0.08	0.58 (0.32–1.05)	0.08	0.57 (0.30–1.06)	100	24.8	277	37.6	<0.01	0.55 (0.42–0.72)	˂0.001	0.49 (0.37–0.65)	˂0.001
Embryo score composite ^d^	7.05 ± 2.01		5.63 ± 2.65		0.001	−2.05-−0.79	0.001	1.26 (1.09–1.45)	6.88 ± 1.96		5.53 ± 2.50		<0.001	−1.61–−1.09	˂ 0.001	1.26 (1.18–1.34)	0.45
Endometrium thickness (mm)	11.44 ± 2.20		11.64 ± 2.68		0.59	−0.52–0.91	0.77	0.98 (0.87–1.11)	11.72 ± 2.49		11.67 ± 2.45		0.75	−0.35–0.25	0.83	0.99 (0.94–1.05)	0.42
Age of male partner (years)	36.70 ± 5.93		37.60 ± 5.47		0.27	−0.69–2.47	0.38	1.03 (0.97–1.10)	37.50 ± 5.80		38.80 ± 6.20		0.001	0.56–2.05	0.61	1.01 (0.18–1.03)	0.03
Sperm parameters																	
Concentration (×10^6^/mL)	71.99 ± 44.56		67.79 ± 39.18		0.47	−15.73–7.34	0.58	1.00 (0.10–1.1)	47.32 ± 40.88		43.16 ± 41.19		0.10	−9.15–0.84	0.46	1.00 (1.00–1.01)	˂0.001
Normal sperm morphology (%)	49.15 ± 16.638		51.36 ± 16.54		0.35	−2.44–6.87	0.20	0.99 (0.97–1.01)	43.70 ± 18.63		41.45 ± 18.67		0.05	−4.52–−0.02	0.08	1.01 (1.00–1.01)	˂0.001
Normal sperm motility > 4% (*n*; %)	60	80	123	85.4	0.21	0.65 (0.34–1.21)	0.35	0.69 (0.32–1.50)	248	61.5	437	59.3	0.49	1.10 (0.86–1.41)	0.51	1.09 (0.84–1.41)	0.75
Normospermia (*n*; %)	52	69.3	112	77.8	0.19	0.65 (0.34–1.21)	0.21	0.66 (0.34–1.27)	202	50.1	316	42.9	0.02	1.34 (1.05–1.71)	0.03	1.32 (1.02–1.70)	0.10

Continuous variables are displayed as mean ± standard deviation (SD) while categorical variables are presented as *n*; %. BMI = body mass index; CI = confidence interval; IVF = in vitro fertilization; ICSI = intracytoplasmic sperm injection; OR = odds ratio; 95% CI = confidence interval; SD = standard deviation; FSH = follicle stimulating hormone; LH = luteinizing hormone; AMH = anti-Müllerian hormone. ^a^ *p*-value, OR, and 95% CI for comparison of categorical data with Fisher’s exact test or chi-square test for categorical variables, and *p*-value and 95% CI for independent sample *t*-tests for continuous variables.^b^ *p*-value for the multivariate linear regression for continuous variables and binomial logistic regression for dichotomous variables. Adjusted odds ratio (AOR) was provided for logistic regression. All variables were adjusted for age, body mass index, previous number of IVF-cycles and double embryo transfer status. ^c^ *p*-value for the Mantel–Haenszel test for categorical variables and two-way ANOVA for continuous variables. ^d^ Embryo score composite: SET with “poor” embryo quality was marked with 1; DET with “poor” + “poor” was marked with 2; DET with “poor” + “good” was marked with 3; DET with “poor” + “excellent” was rated as 4; SET with “good” was marked with 5; DET with “good” + “good” was categorized as 6, SET with excellent was interpreted by 7 and DET with “excellent” + “excellent” was displayed as category 8.

**Table 2:** Predictors of clinical pregnancy for women with only tubal factor infertility vs. non-tubal infertility conceived IVF/ICSI at the Institute of Reproductive Medicine, Albert Szent-Györgyi Medical Centre, University of Szeged, Szeged, Hungary between 1 January 2020 and 30 September 2023 regarding clinical pregnancy (*n* = 1359).

Variable	Only Tubal Factor Infertility (*n* = 219)			Variable	Non-Tubal Factor Infertility (*n* = 1140)		
	*p*-Value	AOR (95% CI) ^a^	B ^a^		*p*-Value	AOR (95% CI) ^a^	B ^a^
Maternal age	˂0.001	0.87 (0.81–0.93)	−0.142	Maternal age	˂0.001	0.92 (0.90–0.95)	−0.079
Embryo score composite *	0.003	1.24 (1.07–1.42)	0.212	Number of previous IVF/ICSI cycles	0.042	0.89 (0.79–0.99)	−0.118
				SET vs. DET	0.007	0.67 (0.50–0.90)	−0.460
				Embryo score composite *	˂0.001	1.26 (1.18–1.34)	0.228
				FSH level	0.004	0.93 (0.88–0.98)	−0.074

FSH level, number of follicles and AMH (anti-Müllerian hormone) level are not significant prognosticators due to these factors interfering with the maternal age and other significant univariate factors. Paternal age and the rate of normospermia does not come to prominence due to their correlation with other factors, mostly normozoospermia. IVF = in vitro fertilization; ICSI = intracytoplasmic sperm injection; AOR = adjusted odds ratio; 95% CI = confidence interval; SET = single embryo transfer; DET = double embryo transfer, FSH = follicle-stimulating hormone. **^a^** *p*-value, AOR, 95% CI and unstandardized B coefficient for binary logistic regression model were provided. All variables were adjusted for age, body mass index, previous number of IVF-cycles and double embryo transfer status. * Embryo score composite: SET was coded as poor (1), good (2) or excellent (3) quality depending on the transferred embryo quality. Furthermore, in case of DET, poor + poor embryos were scored 4, poor + good quality embryo was marked with 5, etc. DET scores ranged from four to nine.

## Data Availability

The data can be made available by the corresponding author on request.
